# COMmunity PARticipation through Education (COMPARE): effectiveness of supported education for students with mental health problems, a mixed methods study – study protocol for a randomized controlled trial

**DOI:** 10.1186/s12888-021-03329-5

**Published:** 2021-07-03

**Authors:** Jacomijn Hofstra, Jorien van der Velde, Petra Jannette Havinga, Lies Korevaar

**Affiliations:** Research and Innovation Centre for Rehabilitation, Hanze University of Applied Sciences, Zernikeplein 23, 9747 AS Groningen, The Netherlands

**Keywords:** Supported education, Students, Mental health problems, Randomized controlled trial, Mixed methods study

## Abstract

**Background:**

The onset of mental health problems generally occurs between the ages of 16 and 23 – the years in which young people follow post-secondary education, which is a major channel in our society to prepare for a career and enhance life goals. Several studies have shown that students with mental health problems have a higher chance of early school leaving. Supported Education services have been developed to support students with mental health problems to remain at school. The current project aims to study the effect of an individually tailored Supported Education intervention on remaining at school, study success, and satisfaction of students with mental health problems studying at an institute for intermediate vocational education and a university of applied sciences in the Netherlands.

**Methods/design:**

The design combines quantitative research (Randomized Controlled Trial; RCT) with qualitative research (monitoring, interviews, focus groups). One hundred students with mental health problems recruited from the two educational institutes will be randomly allocated to either the intervention or control condition. The students in the intervention condition receive the Supported Education intervention given by a Supported Education specialist, the students in the active control condition receive support as usual plus advice from a trained staff member on potential supportive resources regarding studying with mental health problems. The primary outcome ‘remaining at school’, and the secondary outcome ‘study success’ will be determined using data from the school’s administration. The secondary outcome ‘student satisfaction’ and other variables that will be studied in a more exploratory way, such as self-efficacy and study skills, will be determined through online questionnaires at baseline, at 6 and at 12 months follow-up. Focus groups and interviews with the students and Supported Education specialists will be carried out to complement the trial.

**Discussion:**

This RCT is the first to assess the effect of Supported Education on remaining at school, next to study success and student satisfaction among students with mental health problems. The use of a mixed-methods design will result in a thorough evaluation of the effect of the intervention. Issues regarding the influx and possible attrition of students in the follow-up are discussed.

**Trial registration:**

The study was registered with Trialregister.nl, no. NL8349, date registered: February 4th 2020. Register name: Community participation through education. Effectiveness of Supported Education for youth with mental health problems, a mixed methods study – Study protocol for a Randomized Controlled Trial.

Protocol Version: 3, date: May 28th, 2021.

**Supplementary Information:**

The online version contains supplementary material available at 10.1186/s12888-021-03329-5.

## Background

Over the past decade, an increase in mental health problems is seen among students in post-secondary education worldwide and these mental health problems are found to negatively influence the educational goals of these students [1–7]. For instance, having mental health problems increases the risk of dropping out of vocational and higher education up to twice as high [8–11]. For example, in the study of Hjorth et al. [9], the dropout rate amongst students with mental health problems was approximately 15% after five years, compared to a dropout rate of 8% in students without mental health problems. In an economy that requires (higher) education for upward occupational mobility, people who are unable to succeed in post-secondary/higher education or training may find themselves ultimately underemployed or unemployed [12–14]. Therefore, it is of the utmost importance to support students with mental health problems to complete their studies. Supported Education could be the support that is needed to reach that goal.

Supported Education (SEd) is defined as the provision of individualized, practical support and instruction to assist people with mental health problems to choose, get and keep their educational goals [15–18]. The mission of SEd is derived from the choose-get-keep model of the Boston Psychiatric Rehabilitation Approach (PRA): To help people with mental health problems develop the skills and supports required to be successful and satisfied in their chosen roles or environments [15, 19].

A SEd toolkit, based on this choose-get-keep model of psychiatric rehabilitation has already been developed to support students with mental health problems to return to or to remain at school. This toolkit provides tools and guidelines for organizations and professionals working with people with mental health problems [20]. In a small pilot project, the ‘keeping phase’ of the toolkit has been tested among eleven professionals and eleven students with mental health problems who follow post-secondary education [21]. The goal of the ‘keeping phase’ is to remain at school and increase study success and student satisfaction through the development of skills and support. The first results were positive: professionals indicated that the tools were useful, concrete and user-friendly; the students indicated that they felt more supported, got higher grades, and that their school functioning in general improved.

In the current project, we aim to test the effectiveness of the ‘keeping phase’ of the SEd toolkit more extensively and systematically using a randomized controlled trial (RCT) at an institute for intermediate vocational education and a university of applied sciences in the Netherlands. This RCT will be the first to assess the effect of the individualized SEd intervention on ‘remaining at school’ (primary outcome), next to indicators of study success and student satisfaction. These outcomes were chosen as remaining at school, and increasing study success and student satisfaction are the goals of SEd, and increasing the capacity of people to be successful and satisfied in the living, working, learning, and social environments of their choice is also the mission of PRA of which SEd is derived. Student satisfaction is strongly related to study success [22], and to involuntary dropout (i.e. students who were dismissed for failure to meet minimum academic standards) [23]. Furthermore, student satisfaction also influences students’ decision to continue with or drop out of school [24] even in students who were in good academic standing, but choose to dropout [23]. Examining whether the SEd intervention positively influences student satisfaction is therefore of high relevance. We are aware of only three RCTs on the effectiveness of SEd [25–27]. These studies differ from our RCT on several aspects: the type and contents of the intervention, the target group and the outcomes. Collins and colleagues [25] and Mowbray and colleagues [27] studied the effect of two *group* SEd interventions: a classroom intervention and a group model intervention that were more aimed at preparing for going to school, than at remaining at school. Collins and colleagues [25] found that students in the classroom model showed higher levels of empowerment and school self-efficacy than students in the control condition. Additionally, they found that the level of satisfaction with the program of the students receiving the group model intervention was significantly higher than of the students in the control condition. Mowbray and colleagues [27] only performed within-group analyses, so no conclusions can be drawn about the differences on the outcome variables between the experimental condition and the control condition. An RCT by Ellison and colleagues [26] was performed among *veterans* with post-traumatic stress disorder and showed that veterans in the SEd condition spent a greater amount of time on educational activities than the veterans in the control condition. In addition, several *pilot* studies on SEd have been performed (see for an overview of studies published between 1989 and 2009: [28], and for an overview of studies published between 2010 and 2020: Büttner S, Hofstra J, Farkas M, van der Velde J, Korevaar EL: Supported Education for students with mental health conditions: a systematic review of effectiveness studies from 2009-2020, submitted), showing positive effects on for instance educational status and level of employment [29, 30], study enrollment [31, 32], and Grade Point Average [29, 32]. However, the lack of a randomized controlled group in these studies limits the ability to draw strong conclusions. Although existing studies indicate that SEd may be an effective tool to support students with mental health problems, the outcome variables that were studied were very diverse and it remains to be determined whether SEd increases the chance that students remain at school, which is the ultimate goal of the keeping phase of SEd. If SEd is to become a viable alternative and widespread intervention and if mental health and education policies are to emphasize educational attainment, more effectiveness research on SEd models is critically needed [28, 33]. Therefore, in the current study we aim to examine the effect of SEd on remaining at school, next to study success and student satisfaction, using a mixed-methods design. The effect of SEd will be compared with the effect of an active control condition in which students will receive support as usual plus advice from a trained educational staff member on potential supportive resources. We chose for an active control condition to make sure that potential differences in effects of the intervention and control group cannot be attributed to general effects of receiving support, like getting attention from professionals, or being part of a group. Moreover, we thought it to be unethical to not support students who indicate that they are in a current need for support with their school functioning because of their mental health problems.

This design combines quantitative research (RCT) with a process evaluation through qualitative research (monitoring, interviews, focus groups). This process evaluation will be conducted to gain insight into the fidelity, reach, students’ experiences, experiences of the support staff of both the intervention and control condition, success- and fail factors, essential components of the SEd intervention according to students and SEd specialists and recommendations to further adapt the SEd intervention. As part of our examination of essential components of the SEd intervention, we will examine which of the five principles ([20, 34], Mullen MG, Nemec PB, Sullivan-Soydon AP, Thompson JL, Ellison ML, Sabella K, Stone B, Banko AL: Helping youth on the path to employment manual, unpublished; Sullivan-Soydon AP, Legere L: Supported Education operations manual, unpublished) of the choose-get-keep model of PRA [15, 19] on which the SEd intervention is based, are regarded as most helpful to achieve the students’ educational goals.

The principles of the choose-get-keep model of PRA are:

- Self-determination: students make their own choices (setting their own educational goals) and accept responsibility for their educational process.
- Students are actively involved in their SEd process, determining the criteria for success and satisfaction, as well as in evaluating progress toward meeting their goals.
- Partnership between participant and SEd specialist.
- Development of participant skills and of environmental support/resources
- Support as long as needed and wanted

To our knowledge, the importance of these principles according to the users of the SEd intervention has not been evaluated before.

### Research aims

This study involves an effect evaluation (RCT) and a process evaluation (qualitative research) of SEd. The first question that will be addressed is:

A) What is the effect of the SEd intervention on remaining at school (primary outcome), study success and student satisfaction (secondary outcomes) of students with mental health problems who follow post-secondary education, as compared to students with mental health problems who receive support as usual plus advice from a trained educational staff member on potential supportive resources?

We plan to test the following hypotheses:

1. More students who receive the SEd intervention will remain at school or graduate as compared to students who receive support as usual plus advice on potential supportive resources.
2. Students who receive the SEd intervention will be more successful at school, operationalized as a higher GPA and a higher number of credits and courses completed, than students who receive support as usual plus advice on potential supportive resources.
3. Students who receive the SEd intervention will experience a higher level of satisfaction at school than students who receive support as usual plus advice on potential supportive resources.

The second research question that will be addressed is:

B) What are the essential components of the SEd intervention according to students and SEd specialists?

## Methods/ design

### Design

This study uses a mixed methods design that combines quantitative research (RCT) with qualitative research (monitoring, interviews, focus groups), including an intervention condition and an active control condition. The students in the intervention condition receive the SEd intervention, the students in the active control condition receive support as usual plus advice from a trained educational staff member on potential supportive resources regarding studying with mental health problems.

### Participants

Study participants are students with mental health problems who want to complete their current education studying at Mbo Utrecht, which is an educational institute that provides post-secondary vocational education, or at the Hanze University of Applied Sciences Groningen, an educational institute for higher professional education. They will be recruited through personal contact with educational professionals, information brochures (see Additional file 1), posts on the internal website of the schools, posters and social media. Inclusion criteria are:

1. having a mental health condition (e.g. depression, anxiety disorder, bipolar disorder, psychosis, ADHD) for which the student has received a formal diagnosis or the student experiences symptoms for at least 6 months, and;
2. receiving treatment for mental health problems or having received treatment in the past, and;
3. being at least 16 years of age, and;
4. following regular post-secondary vocational education in Utrecht; or higher education in Groningen, and;
5. expressing the need for extra support to remain at school.

Exclusion criteria are:

1. not fluently speaking and reading in Dutch, or;
2. the need for extra support is only expressed by others than the student, or;
3. unable to give informed consent, or;
4. less than six months to go before graduation, or;
5. has received SEd services in the past

### Sample size calculation

As mentioned before, this is the first RCT on the effect of our SEd intervention on remaining at school, our main outcome measure. Therefore, the effect size of our intervention on remaining at school is unknown. In such cases, it is a common convention to set the effect size at 0.5 [35], because a lower effect size would not be considered as clinically relevant [36, 37]. Therefore, we aim to demonstrate an effect size of 0.5 on the primary outcome measure remaining at school with a power of 80% and an alpha < 0.05.

Hence, for each condition 51 students are needed (total 102). Given an expected drop-out percentage of 25% from the study, 140 students (2 × 70) need to be included in the study. This is a feasible number, considering that 20–45% of students worldwide experience mental health problems [1, 38, 39].

### Study procedures

We adhere to the SPIRIT 2013 guidelines [40], the checklist is provided in the Additional file 2.

Students who heard about the study through one of the recruitment strategies and are interested in participation, contact the research team by e-mail. A data coordinator will check the inclusion criteria and will then send a link to an online questionnaire which starts with information about the study and the question to fill out an informed consent form. In the informed consent (see Additional file 3), students state that they have been informed about the study and that they participate voluntarily. In addition, they give permission to collect grades and course credits from the school’s administration for the next five years, so we are able to follow the study success of the students after the current project has finished. They also give permission that in case they drop-out of the study, school progress keeps being monitored. Students have the right to withdraw this permission at any moment in time.

After signing the informed consent, the students fill out the baseline-questionnaire. Then, students will receive a student ID number and will be randomized, for both educational institutes separately, by the data coordinator in either the experimental condition (Supported Education-coaching; half of the group of students) or the control condition (support as usual plus advice on potential resources; half of the group of students), using the minimization approach [41] which aims to minimize imbalance between the conditions on specific factors, in our case on gender, age, ethnicity, study year, level of study, the number of credits completed, and level of psychological distress. The condition to which the student is allocated depends on the characteristics of the students that were already enrolled. Students will receive compensation (a gift coupon of 12,50 euro each) for filling in the questionnaires at baseline, 6- and 12-month follow-up. Within this 12-month follow up, each student experiences a critical moment in the educational pipeline: either transitioning to the next year or graduating. In Table 1, the Spirit flow diagram of the study procedure is presented.

**Table 1:** SPIRIT flow diagram of the study procedure showing enrolment, interventions, and assessments

	Pre-intervention	Post-intervention (within one month)	6 month follow-up	12 month follow-up
**TIMEPOINT**	**t**_**0**_		***t***_***1***_	***t***_***2***_
**ENROLMENT**				
Eligibility screen	X			
Informed consent	X			
Allocation	X			
**INTERVENTIONS**				
*Supported Education - coaching*			X	X
*Control intervention*			X	X
**ASSESSMENTS**				
**Demographics**				
*Age*				
*Gender*				
*Cultural background*	X		X	X
*Diagnosis (and age of onset)*				
*Treatment*				
*Medication use and type*				
*Type, year and level of education*				
*Hours spent on education*	X		X	X
*Employment*				
**Remaining at school**			X	X
**Student success**				
*(GPA, number of courses and credits completed)*	X		X	X
**Student satisfaction**				
*The students’ life satisfaction questionnaire – school subscale*	X		X	X
**Other measures**				
*List with study skills*	X		X	X
*List with resources*	X		X	X
*The General Self-Efficacy scale*	X		X	X
*The Working Alliance Inventory*			X	X
**Psychological distress**				
*Symptom Checklist − 10*	X		X	X
**Evaluation**			X	X
*Interview*		X		
*Focus groups*				X
**Fidelity**				
*Interview*			X	

The quantitative data will be collected online for both sites by the data coordinator using online questionnaires through Enalyzer (a secure software program for research purposes). The data in Enalyzer will be linked to the student ID for this study so that the answers are not linked to identifiable information. All data will be handled confidentially in compliance with the Dutch Personal Data Protection Act (Wet Bescherming Persoonsgegevens; WBP). All data will be stored and locked at the Hanze University of Applied Sciences Groningen and will be destroyed ten years after finishing the study.

Students are blinded for which type of intervention is the experimental/control intervention. They are told that we will compare two types of support services of which they will receive one. The data coordinator who will perform the randomization process is obviously not blinded as is the researcher who will conduct the interviews about the support with the students afterwards. However, they have no personal interest in the outcomes.

### Interventions

#### Experimental condition: supported education- coaching

SEd is a tailor made, personalized intervention, taking the individual’s own educational goal as starting point of the intervention and helping the student assessing the personal critical skills and supports to achieve the personal educational goal. It is a manualized psychiatric rehabilitation intervention suitable for different mental health and educational contexts (see [20, 42, 43] for a detailed description of the SEd intervention). The SEd intervention consists of five steps:

1. Helping the student setting his/her own educational goal
2. Identifying the critical skills and support that the student needs to be successful and satisfied at his/her school of preference
3. Writing the Individualized Study Support Plan. This plan defines what the needed and critical skills and support are to be successful and satisfied at school and how to respectively teach and organize them.
4. Teaching the student the critical skills (cognitive, social and emotional)
5. Organizing the critical support (people, places, activities, things and accommodations)

The student has regular meetings with the SEd specialist. These meetings take place in person, online or by telephone, depending on the preference and the schedule of the student.

The SEd specialist in this study is an educational professional who has been trained for at least three days in the ‘keep phase’ of SEd as described in the Handbook Supported Education [42].

#### Control condition: support as usual plus advice on potential supportive resources

Students in the control condition get support as usual plus advice on potential supportive resources. At Mbo Utrecht, students in this condition meet on a regular basis with their own student service provider who advices them about regular supportive resources that are in place at the educational institute. At the Hanze University, there are no student service providers with comparable tasks, therefore an educational staff member has been trained on giving advice to students about potential supportive resources. In three face-to-face meetings and two consults by phone, this staff member provides the students in this condition with:

- Information on formal and informal support opportunities inside and outside the school (e.g., contact with a fellow student or a family member or a consult with a disability support service member or a psychologist)
- Basic information on studying with mental health problems (e.g., rights and responsibilities of students, students’ stories of studying with mental health problems)
- Information on assistive technology and sources

Subsequently, the student decides for himself whether he follows the advice of the staff member. It is recorded by the student service providers (at Mbo Utrecht) and the staff member (at the Hanze university) how many meetings take place and what kind of support is offered.

### Measurements

A complete list of measurements and measure points can be found in the SPIRIT flow diagram (Table 1). At baseline and the 6- and 12-month follow-up, the following demographic information will be collected: age, gender, cultural background, type, year and level of education, number of hours spent on education per week (i.e., school hours and homework), employment information (number of hours), diagnosis (including age of onset), treatment, medication use and type of medication, and psychological distress. The latter will be measured with the Hopkins Symptom Checklist-10 [44, 45], which is an abbreviated version of the widely used SCL-90. The SCL-90 has been validated in the Dutch language [46]. The items used in the SCL-10 are derived from this validated Dutch translation of the SCL-90, and proved to perform almost as well as another abbreviated version of the SCL-90, the SCL-25 [47]. In the study of Strand et al. [47] a large group of young adults (aged 18–24 years) participated (*n* = 877). On a 5-point scale ranging from 1 (not at all) to 5 (a lot) students indicate whether they have been hindered by symptoms or problems such as ‘Feeling tense’ and ‘Feelings of worthlessness’. The SCL-10 is included in order to describe and confirm the presence of symptoms in the population of the study and is an addition to the inclusion criteria of having a formal diagnosis or experiencing symptoms for at least six months, and receiving treatment for mental health problems or having received treatment in the past. Many research studies that do not administer symptoms checklists are speculated to recruit individuals who do not have “serious or persistent mental illnesses”. Such allegations reduce the study’s ability to generalize to the larger population of people with mental illnesses. Furthermore, this checklist will be used to verify that SEd does not have a negative impact on psychiatric symptoms and level of distress.

#### Primary study outcomes

The main outcome measure will be ‘remaining at school’. At the 6- and 12-month follow-up, it will be examined whether the student remained at school (or graduated) or has dropped-out. If students dropped-out of the study, the reason for dropping-out (voluntary, involuntary, graduation) will be checked.

#### Secondary study outcomes

Secondary study outcomes are study success and student satisfaction.

*Study success* will be measured with the following indicators: grade point average per semester; number of credits completed per semester; and number of courses completed.

*Student satisfaction* will be determined by the school subscale of the Multidimensional students’ life satisfaction scale [48, 49], which consists of eight items measuring the overall satisfaction of the student at school. Students indicate on a 4-point scale, ranging from 1 (Rarely) to 4 (Always) to what extent the statements, such as ‘I like the activities at school’ and ‘I learn a lot at school’ are applicable to them.

Furthermore, the following measures will be included in order to examine whether the scores of the students in the two conditions differ on these aspects which could account for any possible effects:

- The General Self-Efficacy Scale [50, 51], which is a 10-item instrument to examine a person’s belief that his or her actions are responsible for successful outcomes, that is, that they have control over the demands of the environment. Students are asked to indicate on a 4-point scale ranging from 1 (Not at all true) to 4 (Exactly true) to what extent the items are applicable to them. Sample items are ‘ It is easy for me to stick to my aims and accomplish my goals’ and ‘When I am confronted with a problem, I can usually find several solutions’. The psychometric properties of this instrument are examined among 19,120 (young) adults from 25 countries, including the Netherlands, and proved to be good [51].
- The Working Alliance Inventory [52] measures the quality of the working alliance between professional and client with 36 items, divided in three subscales, bond (between professional and client), goals (agreement about the goal of the treatment) and tasks (agreement about the tasks of the treatment and the responsibilities to perform these tasks). Students are asked to indicate on a 5-point scale ranging from 1 (never) to 5 (always) to what extent the items are applicable to them. Sample items are: ‘I do not feel at ease with [-----] and ‘[-----] knows exactly what my goals are’. The Working Alliance Inventory has been translated into Dutch and has proven to have good content validity, internal consistency and construct validity among a sample of adults in rehabilitation research [53]. Students who receive the SEd intervention, answer these questions with their SEd specialist in mind; students in the control condition are asked to answer these questions with the student service provider (at Mbo Utrecht) or the educational staff member (at the Hanze university of applied sciences) in mind.
- A (self-constructed) checklist with 50 study skills (e.g. making notes; preparing for exams, and concentrating) which we constructed ourselves and was inspired on [54]. The student is asked to indicate for each of the study skills whether he can perform that skill or has difficulty with it. If he indicates that he has difficulty performing the skill, he is asked to indicate whether he needs support with it or already receives support with performing the skill. The student has the option of adding study skills that were not mentioned in the checklist (see Additional file 4).
- A checklist with 47 possible formal and informal resources, inside and outside the educational setting (e.g. study advisor; quiet room to study; note book) which was also self-constructed (see Additional file 4). The checklist consists of all available resources at the two sites, complemented with resources that are often mentioned by students in the resource assessment that we apply during SEd trajectories [42]. The student is asked to indicate which of the listed resources he needs and whether he already uses that resource or not. The student has the option of adding resources that were not mentioned in the checklist.

Both checklists are used to give a descriptive indication of the number of study skills with which students experience difficulties and the number of resources they use and need.

In addition, the number and duration of contacts with the professional (SEd specialist or the student service provider or the trained staff member) will be registered and it will be determined whether the two conditions differ in this respect.

### Proposed analyses

Analyses will be performed according to the intention-to-treat principle and we will control for any variation in observable characteristics between conditions that remained after the minimization procedure that was used to randomize the students into the conditions. Missing values in the questionnaires will be prevented as the questionnaires are administered through an online secure software program (Enalyzer) in which answering to all items is obligated. The main outcome measure (remaining at school) will be insensitive for missing values as this data can be retrieved from the administration offices of the educational institutes. In case of missing data (for example, when a student does not fill in the second or third questionnaire), intention-to-treat analyses will be performed to account for incomplete outcome data [55]. In addition, it will be examined whether the rates of incomplete data differ between the control and experimental condition. To detect any outliers, the median absolute deviation method will be used [56]. Unrealistic outliers (due to e.g. data entry mistake) will be removed. In case of outliers with a realistic value, we will perform the analyses both with and without the outliers and we will report both results.

Descriptive statistics (e.g. frequencies, means and standard deviations) will be provided for all variables. Conventional statistical tests (e.g. regression, chi-square and ANOVA) will be used to address the research questions in accordance with how the data align with the assumptions of these tests (e.g. normality, homogeneity). In case the data are not normally distributed, the non-parametric equivalents will be used (e.g. the Friedman-test, the Mann-Whitney-test and the Wilcoxon signed-rank test).

With respect to the primary outcome, remaining at school, we will compare the number of students who remained in education or graduated in the experimental and the control condition at T2. Logistic regression models will be conducted to test the differences between the experimental and control condition. In addition, the differences in scores on the secondary outcomes, study success and student satisfaction, will be examined for T0 to T2 using chi squares or repeated measures of variance with the ANOVA test using 95% confidence levels, respectively. Furthermore, the differences between the two conditions on number and duration of contacts with the SEd specialist, the trained staff member or students service provider; the number of study skills where the students need support with and the number of resources needed or used when studying, student general self-efficacy levels and the working alliance with the professional, will be calculated using the ANOVA test with 95% confidence levels. Moreover, to test whether outcomes are similar for students from both educational institutions, sensitivity analyses will be performed excluding students from the institute for intermediate vocational education.

### Process evaluation

The process evaluation focuses on five main questions recommended by the guideline for process evaluation of Movisie (the Dutch national knowledge institute in the social domain [57]). These five main questions are: 1) Was the intervention conducted as planned? 2) Wat is the reach of the intervention? 3) What are the experiences of participants and support staff? 4) What are the success and failure factors of the intervention? 5) What recommendations can be made based on the process evaluation to improve the intervention? The process evaluation will be conducted for both sites separately (i.e., Groningen and Utrecht). Table 2 shows how the five process evaluation questions will be addressed in the present study.

**Table 2:** Overview of instruments used to address the five process evaluation questions

Main topic	Experimental condition	Control condition
	*Instrument (timing)*	*Instrument (timing)*
1. Fidelity	SEd fidelity questionnaire (T1)	Evaluation checklist (after each meeting)
2. Reach	Registration information: - number of students who contacted the research team to participate in this study - number of students who eventually participated in the trial - recruitment period - number of drop-outs	Registration information: - number of students who contacted the research team to participate in this study - number of students who eventually participated in the trial - recruitment period - number of drop-outs
3a. Students’ experiences	- Interview (within one month post-intervention) - Drop-out interview (within one month after drop-out) - Focus group (T2)	- Questionnaire (T2) - Drop-out interview (within one month after drop-out)
3b. Experiences of support staff	- Evaluation checklist (after each meeting) - Interview (T2)	- Evaluation checklist (after each meeting) - Interview (T2)
4. Succes and failure factors (including essential components^a^)	Based on the outcomes of 1–3.b	Based on the outcomes of 1–3.b
5. Recommendations	Based on the outcomes of 1–4	Based on the outcomes of 1–4

^a^Part of the evaluation of the experimental condition

#### Fidelity

To assess fidelity, the SEd specialists will be interviewed once about one of their –randomly chosen- students, using a SEd fidelity questionnaire which is based on the Psychiatric Rehabilitation (PR) fidelity questionnaire used in a RCT on PR [58, 59]. The PR fidelity questionnaire was originally developed in Dutch and is aimed at four life areas: living, working, learning and socializing. For our SEd fidelity questionnaire we only used the questions about ‘learning’. With this questionnaire, the degree to which the SEd intervention is adhered to, will be scored. A score of 70% or higher will be regarded as sufficient fidelity [60].

The staff member in the control condition will fill out a short (self-constructed) evaluation checklist on which they indicate how they experienced the communication with the student, what they have discussed and, what the structure of the meeting was.

#### Reach

To assess the reach of the study the following data will be recorded: the number of students who contacted the research team to participate in this study, the number of students who eventually participated in the trial, the recruitment period and the number of students who drop out early.

#### Students’ experiences with the interventions

Students in the experimental condition will be interviewed by the researcher about their experiences with the SEd-intervention (semi-structured interview). Interviews will be scheduled within one month post-intervention. The themes covered in the interview are (1) benefits of the intervention (e.g. ‘Did the support help you to reduce the barriers that you experience with studying due to your mental health problems?’), (2) aspects concerning the structure of the intervention (e.g. ‘What did you think of the duration of the appointments?’), (3) materials (e.g. ‘Did you make use of the worksheets? If so, in what way/how did you use them?’), (4) the SEd-specialist (e.g. ‘What did you think of the SEd-specialist?’) and, (5) success- and failure factors, including essential components. (e.g. Students will be asked to indicate for each of the five principles of SEd whether they recognize the principle and which of the principles they regarded as most helpful). Students who drop-out before the study is closed will also be interviewed. This interview covers the same topics as the regular interview, but with an additional topic on reasons for drop-out.

Furthermore, focus groups will be organized at T2 at both sites with students who participated in the experimental condition and thus received SEd. The goal of the focus group is to get more in-depth insight into the essential components of the SEd intervention, as through the interaction with others, students can nuance their thoughts about the intervention. Results from the interviews that took place within one month after ending the support, will be used to determine the content of the focus groups. We plan to conduct two focus groups with six to eight students, one at each site, each focus group will last one-and-a-half to two hours. The focus group will be led by the researcher and a research assistant.

Students’ experiences with the control condition will be examined using a self-report questionnaire (self-constructed) administered at T2. The questionnaire consists of open-ended and closed-ended questions and includes the following topics: (1) benefits of support (e.g., ‘Did the support help you to reduce the barriers that you experience with studying due to your mental health problems?’ Students rate their level of agreement on a scale from 1 = strongly disagree, to 5 = strongly agree), (2) aspects concerning the structure of the support (i.e., ‘What do you think of the number of contact moments you had with the staff member?’. Answer options: 1 = too little, 2 = about right, 3 = too much), (3) material (i.e., ‘The brochure was clear’; students rate their level of agreement on a scale from 1 = strongly disagree, to 5 = strongly agree), (4) the staff member (e.g., ‘What did you think of the staff member?’), and, (5) success and failure factors (e.g., ‘Suppose we start offering this support to students from other colleges or universities as well. What do you think is really important to keep as it currently is?’). Students who drop-out before the study is closed will additionally be interviewed about reasons for drop-out.

#### Experiences of support staff

After each meeting with a student, the SEd specialists will fill out a short (self-constructed) evaluation checklist on which they can indicate how they experienced the communication with the student, which of the five steps of the SEd intervention they have discussed with the student, what they have discussed and what the structure of the meeting was. At T2, the SEd specialists will be interviewed about their experiences with providing the support including what they regard as the essential components. The themes covered in the interview are (1) general information (e.g., ‘How many students did you support using the SEd-intervention?’), (2) support (e.g., ‘Did you feel sufficiently competent to provide the SEd-intervention?’), (3) material (e.g., ‘How did you like working with the worksheets?’), (4) aspects concerning the structure of the intervention (e.g., ‘What did you think of the duration of the appointments?’), (5) benefits of the intervention for students (e.g., ‘What difference did you notice during the SEd-intervention regarding the students’ satisfaction at school?’) and, (6) success- and failure factors, including essential components (e.g., ‘Suppose we start offering this support to students from other colleges as well. What do you think is really important to keep as it currently is?’). The staff members in the control condition will also be interviewed about their experiences at T2 in which the same themes will be covered as in the interview with the SEd specialist, except of course for the question about the essential components of the intervention.

Together, the results of this process evaluation may provide indications for possible improvements in the contents of the SEd intervention or in the implementation of the intervention. In addition, the qualitative results can provide context for the interpretation of the quantitative findings.

### Data analysis and data management

The interviews and focus groups will be audio-taped and transcribed. Subsequently, the data will be analyzed using content analysis techniques [61]. All data gathered in this study will be handled confidentially in compliance with the Dutch Personal Data Protection Act (Wet Bescherming Persoonsgegevens; WBP). All data will be stored and locked at the Hanze University of Applied Sciences Groningen and will be destroyed ten years after finishing the study.

## Discussion

This study will be the first RCT worldwide on the effect of SEd on remaining at school, study success and student satisfaction among students with mental health problems. If the results of this study show positive effects of the SEd intervention on these outcomes, the intervention will be disseminated to mental health and educational organizations, policy makers and to student-, client- and family organizations. In addition, it will be submitted to the database ‘Effective interventions’ of the NJI (Dutch Youth Institute). In this way, also professionals in the field of education and of mental health who did not participate in the project have an effective intervention available to them to support the increasing number of students with mental health problems to remain at school. The use of a mixed-methods design hopefully results in a comprehensive, thorough evaluation of the SEd intervention.

Conducting a RCT brings along a few difficulties. Within the project team, knowledge about RCTs is available and the two sites at which the RCT will be conducted, have experience working with the SEd intervention before. Moreover, an independent advisory board, consisting of students with mental health problems, researchers, and professionals from education and mental health, has been installed at the beginning of the project. This advisory board provides asked and unasked feedback during all phases of the research project. Advisory board meetings will be scheduled twice a year, at which the progress of the study will be discussed, including the trial conduct. Finally, the total procedure (registration, randomization, filling out the questionnaire and the first contact with the Supported Education specialist or the trained staff member in the control condition) has been tested with a student which resulted in a few minor adjustments. For example, the question about receiving treatment has been adjusted so it is clear that treatment for mental health problems is meant and a technical problem with uploading the overview of study progress has been resolved.

A bottleneck of many RCTs is the low influx of participants [62–64]. Therefore, in this study we will be attentive on the inclusion speed: can a sufficient influx of students be expected per site; is there still a willingness to participate at the various levels of the organization, and are disturbing factors for the project expected (e.g. a reorganization)? In addition, we will closely monitor the influx of students during the recruitment phase by evaluating monthly if recruitment rates are on track, in order to identify potential problems in time and initiate additional actions to boost inclusion.

In order to recruit a sufficient number of students, we asked the advisory board to advise us about relevant recruitment strategies. The recruitment strategies that were mentioned like posts on the internal website of the schools and on social media, posters, information brochures and personal contact with educational professionals will be used in the study.

To increase the chance that we reach a sufficient number of students for our study, we decided that the inclusion period of students is six months. Consequently, there will be differences at baseline being the point in the school year the student is enrolled in the study. However, we expect these differences to be the same in the experimental and control condition. We will report this information and if necessary, we will take it into account during the data analysis.

In addition, the attrition of participants can be a threat to studies in which follow-up measurements are used [63, 65]. In the informed consent form of our study, students give permission to collect information about their school progress (drop-out, grades, and course credits) from the school’s administration system also in case they drop-out of the study. In this way, complete and objective data regarding our primary outcome (remaining in school) and a part of our secondary outcomes (study success) are guaranteed.

## Supplementary Information

**Additional file 1. **Model of the information brochure for participants.

**Additional file 2.** SPIRIT 2013 Checklist: Recommended items to address in a clinical trial protocol and related documents.

**Additional file 3. **Model of the informed consent form.

**Additional file 4. **Checklists for 'study skills' and 'support and resources'.

## Data Availability

Not applicable.

## Abbreviations

ADHD: Attention Deficit Hyperactivity Disorder
GPA: Grade Point Average
Hbo: Higher professional education (in Dutch: Hoger beroepsonderwijs)
Mbo: Intermediate post-secondary vocational education (in Dutch: Middelbaar beroepsonderwijs)
PR: Psychiatric Rehabilitation
PRA: Psychiatric Rehabilitation Approach
RCT: Randomized Controlled Trial
SCL-10: Symptom Checklist-10
SEd: Supported Education
SPIRIT: Standard Protocol Items: Recommendations for Interventional Trials
WBP: Personal Data Protection Act (in Dutch: Wet Bescherming Persoonsgegevens)
