# Supplementary material for: COMmunity PARticipation through Education (COMPARE): effectiveness of supported education for students with mental health problems, a mixed methods study – study protocol for a randomized controlled trial
Source: BMC Psychiatry. 2021 Jul 3;21:332. doi: 10.1186/s12888-021-03329-5 (PMC8255018; doi:10.1186/s12888-021-03329-5)
Supplement: Supplementary file 1 — Additional file 1. Model of the information brochure for participants. [file 12888_2021_3329_MOESM1_ESM.docx]

# Model of the information brochure for participants

**COMPARE**

**Supported Education-project**

Support for students with mental health problems

**Information for students**

**Introduction**

If you have mental health problems as a student, this can hinder you in continuing your education. The research centre for Rehabilitation has developed two support methods that we believe will help to reduce the hindrances that you experience. To see if the methods work and to see which method works best, we want to compare them. To this end, we have started the Supported Education project.

**What do the methods look like?**

Two methods have been developed, the Supported Education A-method and the Supported Education B-method. With both methods you will receive support for one year.

The *‘Supported Education A-method’* consists of providing information from whom and where you can get specific support, both within and outside the educational program, in order to maintain your education. During this method, you will receive information about your rights and obligations as a student and about digital and technical resources that are available to support your education. In addition, every three months you will have telephone or face-to-face contact with an staff member about your progress at school. The face-to-face contacts are held at the location where you follow the educational program or at another location if you prefer.

The ‘Supported Education B-method’ consists of individual sessions with a coach. The sessions take place on average once every three weeks and help you identify and realize the skills and resources that are needed to maintain your education. The meetings are held at the location where you follow the educational program or at another location if you prefer.

The participants in the project cannot choose which method they will follow, this is determined by drawing lots. So it depends on coincidence in which group you end up. Both methods are offered for free.

**Research**

In addition to providing support, we also conduct research. With the help of this research we want to find out whether the methods work and which method works best. We ask for your cooperation for this. A total of 50 students from Mbo Utrecht and 50 from Hanze University of Applied Sciences Groningen will be asked to participate in this study.

We ask you to complete a questionnaire three times and to participate in a closing interview. You complete a questionnaire at the start of the study, after six months and after a year at the conclusion of the study. The questionnaire will contain questions about your study progress, your performance at school and how you generally feel. Filling in the questionnaire takes about an hour at a time. At the end of the support there will be a closing interview in which we ask you about your experiences with the method and we will organize a focus group. You will receive compensation for completing the questionnaires and participating in the interview. This will be a gift voucher of € 12.50 three times, so a total of € 37.50. If you participate, we ask you to sign a consent form.

**How are the research data recorded?**

All answers you give to the questionnaires are stored anonymously and are handled encrypted by means of a unique participant number. The results are written up for research journals. Your name does not appear there either. Only the researchers (Lies Korevaar, Petra Havinga, Jacomijn Hofstra and Jorien van der Velde) have access to your personal data, all others involved can only access the anonymised data. The researchers will treat your personal information confidentially and will not share it with others. The information you provide will not be shared with staff from your study program. Sometimes a government body (such as an ethics committee) wants to see whether the researchers are carrying out the research properly. They may therefore view the data. The data will therefore be stored anonymous for 10 years after the research has finished.

**Interested in participation?**

You are eligible to participate if you:

• Have a diagnosis for your mental health problems or if you have been experiencing mental health problems for at least six months, ánd

• You are or have been receiving treatment for your mental health problems ánd

• 16 years or older, ánd

• Follow mainstream education, ánd

• have a need for support with studying ánd

• You still have at least 6 months to go at the study program ánd

• Never had Supported Education support before.

You can register for the project at: [Compare@org.hanze.nl](mailto:Compare@org.hanze.nl)

**What if I do not want to participate anymore?**

Your participation is completely voluntary. If you no longer want to participate, you can stop with the project at any time. You do not have to give a reason for this. Stopping the project has no consequences for following your study progra

**Do you have questions?**

Do you have any questions after reading this information letter, or do you want to know more? Then you can contact us at:

[Compare@org.hanze.nl](mailto:Compare@org.hanze.nl)
