# Supplementary material for: COMmunity PARticipation through Education (COMPARE): effectiveness of supported education for students with mental health problems, a mixed methods study – study protocol for a randomized controlled trial
Source: BMC Psychiatry. 2021 Jul 3;21:332. doi: 10.1186/s12888-021-03329-5 (PMC8255018; doi:10.1186/s12888-021-03329-5)
Supplement: Supplementary file 3 — Additional file 3. Model of the informed consent form. [file 12888_2021_3329_MOESM3_ESM.docx]

# Model of the Informed consent form

| COMPARE – Supported Education project |
| --- |

**Consent statement for participation in the COMPARE study**

Herewith, I

_____________________________________ (Last name and initial(s))

DO/ DO NOT give permission for participation in the study*
**Cross out what does not apply*

If I give my consent, I hereby declare that I have been informed of the nature, method and purpose of the study and that I have been given the opportunity to ask questions. I have had enough time to decide if I wanted to participate in the study.

I voluntarily agree to participate in the study and I give permission for the next 5 years to request my study progress data from the school’s administration. I reserve the right to withdraw this consent without giving a reason for doing so. If I withdraw my permission to participate, I know that I can be referred to the regular support services of my school for help.

Furthermore, I give permission for the collection and use of my data in the manner and for the purposes contained in the information brochure. I give permission to keep the anonymised data for another 10 years after the end of this study.

All information is processed completely anonymously and will not be traceable to me as a person.

Date: _____________________

Signature participant:

___________________________

**To be completed by the researcher:**

I declare that I have fully informed this participant of the aforementioned study.

I am willing to answer emerging questions about the study to the best of my ability.

If information becomes known during the study that could affect the consent of the participant, I will inform him/her in good time.

Name researcher: ­­­­­­­­­­­­­­­­­­­_____________________________

Signature researcher: __________________________
