# Supplementary material for: COMmunity PARticipation through Education (COMPARE): effectiveness of supported education for students with mental health problems, a mixed methods study – study protocol for a randomized controlled trial
Source: BMC Psychiatry. 2021 Jul 3;21:332. doi: 10.1186/s12888-021-03329-5 (PMC8255018; doi:10.1186/s12888-021-03329-5)
Supplement: Supplementary file 4 — Additional file 4. Checklists for 'study skills' and 'support and resources'. [file 12888_2021_3329_MOESM4_ESM.docx]

# Checklists for ‘study skills’ and ‘support and resources’

## Study Skills

Below you will find a list of skills that are related to studying. For each skill, indicate whether you can perform it well or whether you have difficulty with it.

(Answer options: I can perform this/ I have difficulty with this and I receive support for it / I have difficulty with this and don’t receive support for it / N/A). If you are having trouble with the skill, please indicate whether you are already receiving help/support with this. If you have no experience with a skill, please tick 'not applicable'.

1. Transportation to and from school
2. Finding your way at (online) school
3. Using administrative services (like the secretary or STAD)
4. Obtaining financial aid
5. Using school facilities (like the library, learning center)
6. Determining the purpose of assignments
7. Determining which tasks need prioritizing
8. Planning
9. Sticking to your schedule
10. Meeting assignment deadlines
11. Taking notes
12. Completing assignments
13. Memorizing important things
14. Researching information
15. Comprehensive reading
16. Preparing for exams
17. Taking exams
18. Preparing for class
19. Asking questions in (online) class
20. Answering questions in (online) class
21. Working in groups in the (online) classroom
22. Working in groups outside the classroom
23. Giving oral presentation
24. Dealing with internal distractions caused by your own thoughts
25. Reducing distraction by social media
26. Reducing distraction by classmates, noise etc.
27. Making conversation with a teacher/advisor/other employees of the school
28. Dealing with emotions
29. Dealing with emotional themes
30. Responding to other people’s feelings
31. Responding to feedback
32. Giving feedback
33. Making small talk
34. Listening to others
35. Showing understanding
36. Handling free time
37. Participation in students associations or organizations
38. Using social places, like the canteen, at school
39. Finding out what social activities are being organized
40. Participating in organized activities
41. Dealing with social media
42. Asking for help
43. Using support (by persons)
44. Using resources/facilities (such as rooms, arrangements)
45. Speaking with instructors about accommodations that you need
46. Identifying barriers
47. Dropping out of class
48. Requesting a delay
49. Resolving conflicts
50. Organizing a quiet workplace

Are there any skills that are not mentioned, but you do experience difficulty with? Fill this in below. Also indicate whether or not you will receive help/support with this.

----------------------------------------------------------------------------------------------------------------------------------------------------------------------------------------------------------------------------------------------------------------------------

Are there answers you would like to clarify? If so, you can provide this explanation below.

----------------------------------------------------------------------------------------------------------------------------------------------------------------------------------------------------------------------------------------------------------------------------

## Support and resources

During your studies, you can use various types of support and resources to help you study. Below you will find a list of various types of resources/support.

Indicate per type of resource/support whether you need it for studying. If you need this, please indicate if you are already using this form of support/help. (Answer options: I don’t need this type of support/resource – I need this type of support/resource and I already use it – I need this type of support/resource but I don’t use it (yet) – I don’t know this type of support/resource).

You may not be familiar with some types of help/support. If you are unfamiliar with the support/resources or do not know what this person could do for you, you can choose the option "I don't know".

Professionals within the Hanze university

- Study career counselor
- Dean
- Attention officer ‘studying with a disability’
- Hanze student support
- Student psychologist
- Teacher

Are there other professionals within the university from whom you receive or would like to receive help? If so, from whom? Also indicate whether you are already receiving help from them.

---------------------------------------------------------------------------------------------------------------------------------------------------------------------------------------------------------------------------------------------------------------------------

If you add up all the help you get from professionals at the university, how many hours of help do you currently receive?

- Less than 1 hour per month
- 1 to 4 hours per month
- 1 hour a week
- More than 1 hour per week, namely…

Professionals outside of the unniversity who provide support with studying

- Psychologist
- Vocational rehabilitation counselor
- Social worker
- (study)coach

(Answer options: I don’t need this type of support/resource – I need this type of support/resource and I already use it – I need this type of support/resource but I don’t use it (yet) – I don’t know this type of support/resource)

Are there other professionals outside the university from whom you receive or would like to receive help with studying? If so, from whom? Also indicate whether you are already receiving help from them.

----------------------------------------------------------------------------------------------------------------------------------------------------------------------------------------------------------------------------------------------------------------------------

If you add up all the help you get from professionals outside of the university, how many hours of help do you currently receive?

- Less than 1 hour per month
- 1 to 4 hours per month
- 1 hour a week
- More than 1 hour per week, namely…

Other support

- Classmates
- Parent(s)
- Other family members
- Friends

(Answer options: I don’t need this type of support/resource – I need this type of support/resource and I already use it – I need this type of support/resource but I don’t use it (yet) – I don’t know this type of support/resource)

Are there other people from whom you receive or would like to receive help with studying? If so, from whom? Also indicate whether you are already receiving help from them.

----------------------------------------------------------------------------------------------------------------------------------------------------------------------------------------------------------------------------------------------------------------------------

If you add up all the help you get from other people, how many hours of help do you currently receive?

- Less than 1 hour per month
- 1 to 4 hours per month
- 1 hour a week
- More than 1 hour per week, namely…

Places

- Quiet place at home
- Library
- A study place
- Canteen
- Silence room
- A room at a classmates house
- Homework institute
- Other place in the classroom

(Answer options: I don’t need this type of support/resource – I need this type of support/resource and I already use it – I need this type of support/resource but I don’t use it (yet) – I don’t know this type of support/resource)

Are there any other places you need? If so, indicate which one and if you are already using it.

----------------------------------------------------------------------------------------------------------------------------------------------------------------------------------------------------------------------------------------------------------------------------

Activities

- Clear agreements with people
- Walking outside
- Role playing
- Breaks
- Supervision
- Relaxation

(Answer options: I don’t need this type of activity – I need this type of activity and I already use it – I need this type of activity but I don’t use it (yet) – I don’t know this type of activity)

Are there any other activities you need? If so, indicate which one and if you are already using it.

----------------------------------------------------------------------------------------------------------------------------------------------------------------------------------------------------------------------------------------------------------------------------

Things

- Good internet connection
- Mobile with features
- Calculator
- Study book(s)
- Notepad
- Laptop
- Recording device
- Desk
- Alarm clock (separate from mobile)
- Study grant
- Compensation for travel costs
- Medication
- Technological support through apps/computer programs

(Answer options: I don’t need this type of support/resource – I need this type of support/resource and I already use it – I need this type of support/resource but I don’t use it (yet) – I don’t know this type of support/resource)

Are there any other things you need? If so, indicate which one and if you are already using it

----------------------------------------------------------------------------------------------------------------------------------------------------------------------------------------------------------------------------------------------------------------------------

Adjustments

- Extra time on exams
- A private exam room/taking the exam with less people
- Custom exam format
- Custom assignments
- Exemption form compulsory participation
- Adjusted schedule

(Answer options: I don’t need this type of support/resource – I need this type of support/resource and I already use it – I need this type of support/resource but I don’t use it (yet) – I don’t know this type of support/resource)

Are there any other adjustments/facilities you need? If so, indicate which one and if you are already using it.

----------------------------------------------------------------------------------------------------------------------------------------------------------------------------------------------------------------------------------------------------------------------------
